# The Possible Dual Role of the ACE2 Receptor in Asthma and Coronavirus (SARS-CoV2) Infection

**DOI:** 10.3389/fcimb.2020.550571

**Published:** 2020-09-23

**Authors:** Anna Cláudia Calvielli Castelo Branco, Maria Notomi Sato, Ricardo Wesley Alberca

**Affiliations:** ^1^Institute of Biomedical Sciences, University of São Paulo, São Paulo, Brazil; ^2^Laboratory of Dermatology and Immunodeficiencies, LIM-56, Department of Dermatology, School of Medicine and Institute of Tropical Medicine of São Paulo, University of São Paulo, São Paulo, Brazil

**Keywords:** SARS-CoV2, asthma, COVID 19, ACE2, inflammation

A new virus belonging to the *coronaviridae* family has been identified and named severe acute respiratory syndrome coronavirus-2 (SARS-CoV-2) (Zhu et al., 2020). This virus can generate a severe respiratory disease named coronavirus disease-2019 (COVID-19). In November 2019, SARS-CoV-2 began to infect humans and cause high rates of respiratory disorders worldwide; accordingly, COVID-19 was declared a pandemic in March 2020 (Li J. Y. et al., 2020). COVID-19 has already affected millions of people and killed over 600,000 individuals worldwide (WHO, 2020).

Similar to severe acute respiratory syndrome coronavirus-1 (SARS-CoV-1), which was responsible for the 2002 pandemic, SARS-CoV-2 infection is initiated when its S-protein binds to the angiotensin-converting enzyme 2 (ACE2) receptor through which it gains entry into the host's cells (Kuba et al., 2005; Walls et al., 2020). The affinity of SARS-CoV-2 for the ACE2 receptor is 10 times higher than that of SARS-CoV-1 (Wrapp et al., 2020). This receptor is mainly expressed in the lungs and to a lesser degree in other organs, such as the heart, kidneys, and intestines (Bavishi et al., 2020), which could explain the increased prevalence of lung infection.

Many risk factors are associated with the course and severity of COVID-19, including older age, systemic arterial hypertension (SAH), pregnancy, and obesity (Alberca et al., 2020a,b). Although asthma is a debilitating pulmonary syndrome, initial reports have not identified asthmatic patients as having a higher risk for COVID-19 (Guan et al., 2020; Zhang et al., 2020). In this manuscript, we review the possible association between the SARS-CoV-2 entry receptor ACE2 and asthma.

## Asthma

Asthma is a complex respiratory syndrome that affects ~350 million people worldwide (Global Initiative for Asthma, 2018). This disease is generally defined by restricted airflow, airway inflammation, and airway hyperresponsiveness, resulting in symptoms such as shortness of breath, wheezing, and cough; moreover, if untreated, asthma can be lethal (Global Initiative for Asthma, 2018). Asthma mortality seems to be declining worldwide (Ebmeier et al., 2017), and it is estimated that in 2015, over 400,000 deaths occurred due to asthma complications (Soriano et al., 2017). Asthma is also associated with other comorbidities, including SAH (Ferguson et al., 2014) and pulmonary hypertension (Rosival, 1990), which are two established risk factors for COVID-19 (Zhang et al., 2020). Other characteristics associated with the worst asthma symptoms, such as obesity and old age, are also associated with poor COVID-19 prognoses (Schatz et al., 2014; Skloot et al., 2016; Płusa, 2017; Zhang et al., 2020).

Asthmatic patients can suffer from a progressive worsening of symptoms called asthma exacerbation, which necessitates treatment with systemic corticosteroids and eventually mechanical ventilation and intensive care (Dougherty and Fahy, 2009). A common concept in asthma is that viral infections can be associated with asthma exacerbation (Costa et al., 2014), and in respiratory viral infections, asthma patients can upregulate a wide range of molecules expressed in the lungs; one of these molecules is ACE2 (Bai et al., 2015).

## SARS-COV and ACE2

The ACE2 receptor is crucial for COVID-19, as SARS-CoV-2 can only enter ACE2-expressing cells (Zhou et al., 2020). Nevertheless, ACE-2 expression is also important for the control of lung inflammation and damage upon viral infection (Imai et al., 2005; Yang et al., 2014; Zou et al., 2014).

During SARS-CoV-1 infection, the overexpression of ACE2 increases viral infection and replication rate (Li et al., 2003), and in animal models, infection with SARS-CoV-1 is ACE2-dependent (Kuba et al., 2005). However, in SARS-COV-2 infection, Chen et al. proposed a negative association linking ACE2 expression and COVID-19 fatality, as ACE2 expression is reduced in elderly and type II diabetic patients (Yoon et al., 2016; Chen et al., 2020).

Interestingly, children also express low levels of ACE2 in the lungs (Bunyavanich et al., 2020), and the death rate in this group has been described as low (Bialek et al., 2020). In addition to the lower ACE2 expression in the lungs of elderly patients (Wu and McGoogan, 2020), other characteristics, such as the presence of other comorbidities, immune senescence, or low-grade inflammation associated with aging (inflammaging), could influence COVID-19 outcomes (Franceschi and Campisi, 2014; Fuentes et al., 2017).

Another important finding is that men infected with SARS-CoV-2 have more severe disease and higher mortality than women (Sharma et al., 2020). The primary female sex hormone (estrogen), in addition to being able to directly influence immune responses, is able to upregulate the expression of ACE2 (Bukowska et al., 2017).

Recently, it has also been described that some structural variations in the ACE2 receptor can lead to differences in protein binding with SARS-CoV-2, helping to understand different infection profiles in humans and even cases of viral resistance (Hussain et al., 2020). ACE2 may also play a larger role in SARS-CoV-2 infection, participating in postinfection regulation of the immune response, cytokine secretion, and viral genome replication (He et al., 2020).

## Asthma and Covid-19

A report from Wuhan, China, identified a low number of asthmatic patients among COVID-19 patients (0.9%); however, asthma was not associated with greater COVID-19 severity or mortality (Li X. et al., 2020). Another study with 5,700 COVID-19 patients from New York City, USA, identified 479 patients with asthma (9%) (Richardson et al., 2020). In addition to this discrepancy in the incidence of asthma among COVID-19 patients, a recent study with 1,827 patients identified that mortality was similar in asthmatic and non-asthmatic COVID-19 patients (Wang et al., 2020).

Song et al. evaluated the prevalence of asthma and chronic obstructive pulmonary disease (COPD) in patients from a cohort of COVID-19 patients in China and found that 2.3% of the patients had asthma and 2.2% had COPD; none of the patients had asthma and COPD (Song et al., 2020). They verified that COPD patients had a higher risk of severe COVID-19 than asthmatic patients. In addition, the number of ACE2-positive cells in alveolar epithelial cells was lower in asthmatic patients and higher in COPD patients than that in patients without asthma or COPD (Song et al., 2020).

## Asthma, Coronaviruses, and ACE2

Asthma is the most common chronic disease in children (Ferrante and La Grutta, 2018), and in the previous pandemic caused by SARS-CoV-1, asthmatic children infected with SARS-CoV-1 did not sustain an increase in asthma exacerbation (Van Bever et al., 2004). Moreover, a 2019 report indicated that the most common chronic condition in Middle East respiratory syndrome coronavirus (MERS-CoV) patients was asthma (van Kerkhove et al., 2019). In another report, two patients who died from MERS-CoV complications had chronic respiratory syndromes: one had COPD and one had asthma (Min et al., 2016). Therefore, similar viral infections do not present a clear picture of how SARS-CoV-2 infection progresses in asthmatic patients.

In a murine asthma model, ACE2 activation has been implicated in a reduction in airway inflammatory response (Dhawale et al., 2016). It is important to highlight that asthma can be divided into four different endotypes: T helper cell (Th)2 high/eosinophilic, Th17/neutrophilic, Th2/Th17/mixed inflammation, and paucigranulocytic (without an increase in polymorphous nuclear cells in the lungs) (Wenzel, 2013).

The asthma endotype is especially important, as cytokines can modify ACE expression. IL-17 can upregulate ACE2 expression (Song et al., 2020), whereas IL-4 and IL-13 can downregulate ACE2 expression (Kimura et al., 2020; Song et al., 2020).

Eosinophils may also play a larger role in COVID-19, as non-asthmatic patients with COVID-19 who present an absence of eosinophils in the first day of hospitalization have a worst prognose than non-asthmatic patients with eosinophils (Tanni et al., 2020). Another study suggested that eosinophil count in peripheral blood has prognostic value, as patients with a low number of eosinophils were more likely to exhibit shortness of breath and require longer hospitalization time (Xie et al., 2020). An increase in eosinophils is associated with COVID-19 improvement and hospital release (Liu et al., 2020; Xie et al., 2020). We hypothesize that different endotypes of asthma may modify ACE2 expression differently, thereby affecting COVID-19.

ACE2 expression in the lungs is also modulated in other respiratory diseases. In an experimental model of smoke-induced acute respiratory distress, a Th17/neutrophilic syndrome, ACE2 was upregulated (Wösten-Van Asperen et al., 2011). In addition, cytokine release from smoking-associated lung injury induces upregulation of ACE2 in the lungs (Leung et al., 2020). In summary, different endotypes of asthma and patients with multiple characteristics, such as smoking asthmatic patients or asthmatic patients with other morbidities, may also present a difference in lung ACE2 levels.

## Asthma Treatment and Covid-19

Approximately 50–70% of asthmatic patients have Th2 high/eosinophilic asthma (Peters et al., 2014; Seys et al., 2017). Th2 high/eosinophilic asthma can be treated with allergen-specific immunotherapy or symptomatic medication. Allergen-specific immunotherapy is a process that usually increases the circulation of regulatory IL-10-producing cells (Asamoah et al., 2017; Alberca-Custodio et al., 2020), which could help to curb the pro-inflammatory cytokine storm in COVID-19. Asthma medications, such as corticosteroids and long-acting beta agonists, reduce lung inflammation and provide symptomatic control (Asamoah et al., 2017). Recently, the usage of inhaled corticosteroids was also associated with lower expression of ACE2 in the sputum of asthmatic patients (Peters et al., 2020).

Dexamethasone, a long-acting glucocorticoid commonly used in the treatment of asthma exacerbation (Shefrin and Goldman, 2009; Cross et al., 2011), has recently shown positive results in COVID-19 (Recovery Collaborative Group et al., 2020). Dexamethasone treatment reduced mortality among COVID-19 patients receiving respiratory support (RS) but not among patients not receiving RS (Recovery Collaborative Group et al., 2020).

Other anti-asthma drugs (AADs), mainly cromolyn, fenoterol, montelukast, and reproterol, have been postulated to be of potential use in SARS-CoV-2 infection (Wu et al., 2020). These drugs could help reduce inflammation and improve lung function (Mombeini et al., 2012; Davino-Chiovatto et al., 2019). Therefore, both dexamethasone and AAD usage for the treatment of asthma could confer additional protection to asthmatic patients.

Immunobiological treatment, including the use of monoclonal antibodies targeting asthma-related molecules, such as IL-5 and IgE, has proven effective in reducing asthma symptoms (Samitas et al., 2015; Farne et al., 2017). To date, there is no report on the influence of anti-IL-5 on SARS-CoV-2 infection; therefore, the usage of this immunobiological agent during COVID-19 is contraindicated, as this type-2 cytokine could potentially counteract the type-1 cytokines released during infection (Vultaggio et al., 2020).

Interestingly, treatment with anti-IgE decreases endothelin-1 (Zietkowski et al., 2010), and the decrease in endothelin-1 upregulates the expression of ACE2 in bronchial epithelial cells (Zhang et al., 2013). On the other hand, a case of COVID-19 in a patient with severe asthma treated with the anti-IgE antibody did not provide evidence of asthma exacerbation or pneumonia (Lommatzsch et al., 2020). Hence, further studies with a larger cohort are necessary to better understand the role of this immunobiological treatment during COVID-19 and the corresponding influence on the ACE-2 receptor.

## Covid-19 Cytokines, ACE2, and Asthma

SARS-CoV-2 infection can generate a process called a cytokine storm, which is characterized by an increase in the levels of mainly type-1 cytokines, including IL-1, IL-8, IFN-γ, IP10, MCP1, and TNF, in the blood (Huang C. et al., 2020). The concentration of these cytokines can be a predictive factor in a patient's disease course (Huang C. et al., 2020). Investigations on the influence of the interaction between comorbidities, COVID-19 and cytokines on ACE2 expression are crucial for the development of new treatments for COVID-19 (Pagliaro and Penna, 2020). The upregulation of ACE2 is associated with an increase in the levels of IL-1, IL-10, IL-6, and IL-8 (He et al., 2020), which are important cytokines in the pathophysiology of COVID-19 (Guan et al., 2020; Zhang et al., 2020). IL-1 and IL-6 are likely involved in the development of fever, which is the most common COVID-19 symptom (Cartmell et al., 2000; Fabricio et al., 2006). IL-8, or CXCL8, is an important chemokine for the migration of neutrophils to the lungs in acute respiratory distress and the formation of neutrophil extracellular traps in COVID-19 (Wong et al., 2004; Gong et al., 2020; Middleton et al., 2020).

Although ACE2 plays a crucial role in SARS-CoV-2 viral infection, the use of ACE2 inhibitors may not be possible due to ACE2 being a protective factor in acute lung injury (Ye and Liu, 2020). Currently, no specific treatment or vaccine is available for SARS-CoV-2/COVID-19 (Huang L. et al., 2020). A preliminary study by Leng et al. showed that transplantation of seven patients from Beijing YouAn Hospital, China, with ACE2-negative mesenchymal stem cells (MSCs) was effective in improving the clinical outcomes of pneumonia, mainly due to immune modulation, with decreased TNF and increased IL-10 (Leng et al., 2020). Other reports have highlighted the usage of anti-TNF (Brito et al., 2020) and anti-IL-1β to regulate inflammation in COVID-19 patients (Cauchois et al., 2020).

IL-4 and IL-13, which are cytokines highly produced in Th2/eosinophilic asthma, can downregulate ACE2 expression in airway epithelial cells (Kimura et al., 2020; Song et al., 2020), whereas, TNF, IL-12, and IL-17A, which are cytokines highly produced in Th17/neutrophilic asthma and COPD (Alcorn et al., 2009), can upregulate ACE2 expression in *in vitro* BEAS-2B cells (Song et al., 2020). In addition, circulating soluble angiotensin-converting enzyme 2 (sACE2) is upregulated in the blood of asthmatic patients (Ayada et al., 2015); hence, sACE2 could act as a competitive interceptor, limiting SARS-CoV-2 attachment to airway cell membranes (Batlle et al., 2020).

Further investigations are needed to better understand the role of ACE2 in asthmatic patients during SARS-CoV-2 infection, which would enable the development of better and more effective treatments for the COVID-19 pandemic while mitigating deaths in asthmatic individuals and the overall population.

## Author Contributions

AB and MS: write and review. RA: conception, write, and review. All authors contributed to the article and approved the submitted version.

## Conflict of Interest

The authors declare that the research was conducted in the absence of any commercial or financial relationships that could be construed as a potential conflict of interest.
